# Quantifying machine influence over human forecasters

**DOI:** 10.1038/s41598-020-72690-4

**Published:** 2020-09-29

**Authors:** Andrés Abeliuk, Daniel M. Benjamin, Fred Morstatter, Aram Galstyan

**Affiliations:** Information Sciences Institute, University of Southern California, Marina del Rey, CA USA

**Keywords:** Human behaviour, Applied mathematics, Computer science, Information technology

## Abstract

Crowdsourcing human forecasts and machine learning models each show promise in predicting future geopolitical outcomes. Crowdsourcing increases accuracy by pooling knowledge, which mitigates individual errors. On the other hand, advances in machine learning have led to machine models that increase accuracy due to their ability to parameterize and adapt to changing environments. To capitalize on the unique advantages of each method, recent efforts have shown improvements by “hybridizing” forecasts—pairing human forecasters with machine models. This study analyzes the effectiveness of such a hybrid system. In a perfect world, independent reasoning by the forecasters combined with the analytic capabilities of the machine models should complement each other to arrive at an ultimately more accurate forecast. However, well-documented biases describe how humans often mistrust and under-utilize such models in their forecasts. In this work, we present a model that can be used to estimate the trust that humans assign to a machine. We use forecasts made in the absence of machine models as prior beliefs to quantify the weights placed on the models. Our model can be used to uncover other aspects of forecasters’ decision-making processes. We find that forecasters trust the model rarely, in a pattern that suggests they treat machine models similarly to expert advisors, but only the best forecasters trust the models when they can be expected to perform well. We also find that forecasters tend to choose models that conform to their prior beliefs as opposed to anchoring on the model forecast. Our results suggest machine models can improve the judgment of a human pool but highlight the importance of accounting for trust and cognitive biases involved in the human judgment process.

Many high stakes intelligence decisions rely on accurate predictions. Forecasting future political and economic events is notoriously difficult. The forecasts of individuals tend to be inaccurate and unreliable, even if they are experts^1^. When tracked over time, experts exhibit forecasting accuracy not significantly better than a random guess in many domains, including politics^2^. Two solutions have emerged that reliably improve prediction accuracy: eliciting and aggregating forecasts from a pool of forecasters (crowdsourcing) and using machine learning to improve time-series models. Combining even a small number of judgments can lead to stark, stable improvements in accuracy^3^. Statistically aggregating large pools of public forecasts has recently been shown to improve upon individual forecasts^4^. Concurrently, advances in machine learning have led to improvements in feature identification and prediction, provided data is available and favorably structured^5^. Finally, recent work on human–machine ensemble methods shows that aggregating a crowd of forecasts together with machine models can outperform each of the individual components^6,7^.

We present the results of a hybrid system aimed at harnessing the unique benefits of crowdsourcing and machine models. We address open questions about the efficiency of human–machine combinations. Do human forecasters incorporate model predictions in ways that improve their own forecasts, or does bias in their reactions hurt performance? In this work, we study the problem of measuring the influence of machine models on the human-generated forecasts in a hybrid forecasting platform. In order to learn the weight that users assign to machine forecasts, we propose a model that compares the forecasts of users who are exposed to the machines with those who are not. We analyze the weights to uncover patterns about how humans interact with the machine models throughout the course of an eight-month-long forecasting tournament. We show how individuals assign weights, quantify the degree of (dis-)trust in the models, and test the extent to which cognitive biases are prevalent. We conclude by analyzing the impact of trust and biased processing on our systems’ performance.

## Introduction

### Crowdsourcing predictions

Crowdsourcing, a method of engaging many individuals, often via a network, to jointly perform a task, has been demonstrated to solve a variety of innovative and strategic problems^8^. These methods have become popular among organizations because they can perform complex tasks quickly and cheaply. Under the right circumstances, crowdsourcing can achieve a level of collective intelligence when disparate knowledge or skills are combined to perform in seemingly intelligent ways. A successful crowd includes engaged members who are motivated to improve their performance possibly by competing against one another as well as a diversity of appropriate knowledge and expertise^9^. Harnessing a crowd is particularly efficient when the knowledge to solve a certain task is broadly dispersed and not necessarily identifiable in advance^10^.

One common use of crowdsourcing is to forecast future outcomes by collecting forecasts from large groups of individuals about the same outcomes sometimes over a long timeframe. Crowdsourced forecasting requires a system capable of tracking individual performance and motivation over time^9^. Individual inputs can be combined in adept ways including adjusting for detectable biases and leveraging the better forecasters and newer forecasts in the pool^11,12^. In a recent political forecasting tournament ^13^, combining independent forecasts led to substantial improvements in accuracy and reliability. Crowdsourcing can improve upon individual judgments because it: a) amalgamates disparate knowledge, b) cancels individual errors, and c) builds credibility of the group judgment^14^. This “wisdom of the crowd“ (WOC) effect has been demonstrated as a successful approach in diverse domains^15^, even including solving complex, multi-faceted problems^16^. The benefits of such combinations can be achieved with non-expert populations^17^.

### Human versus machine prediction

Recent gains in computational power paired with advances in machine learning and modeling methods suggest that computers may be a suitable alternative to accurately predicting many economic and political outcomes. Machine prediction has advanced from the typical assumptions (e.g., linearity) to create more flexible tools that adjust to changes in the data. Real-time availability of data allows model parameters and predictions to be responsive to data changes. For example, adaptable feature selection and noise reduction techniques help improve upon static models. Indeed, recent works describe such advances in machine-generated prediction accuracy^18^.

Both WOC and machine models have a number of limitations. WOC relies not just on the pool sharing sufficient knowledge about the topic, but also on the diversity of knowledge and expertise which can be difficult to assess. Human pools are also prone to cognitive biases that impact the quality of their judgments. Individual biases like base-rate neglect and overconfidence, as well as group biases like social influence, can negatively impact the judgment of opinion pools^19^. Machine-generated predictions succeed in certain settings, especially when an outcome is autoregressive, but show limited success when causal factors are not well understood or multi-faceted. Other limitations arise from time-series with more difficult structures, such as zero-inflated time-series^20^.

### Developing a hybrid system

Prediction problems that are relevant for political and economic decision-making rely on a complex global system with poorly understood causal structures. Moreover, applicable data sources vary in volatility, structure, and format. Two methods have been proposed for combining judgmental (subjective) and statistical (objective) forecasts: adjustment and combination^21^. We take a “hybrid” approach by building a system aimed at leveraging the strengths of machine models and crowdsourcing to balance generalizability across data sources and problem-types with the flexibility to tackle new, unforeseen problems. We make machine time-series predictions available to human forecasters to help them anchor on a statistical estimate based on historical base rates and recent trends. Empirical tests have found structured combinations of judgmental adjustments to model predictions improve upon time-series models when the data is highly volatile^17^.

Successful hybrid systems have been demonstrated in modeling infectious disease trajectories using social signals^22^ and expert forecasts^23^. The success of such a hybrid approach hinges on whether human forecasters trust and choose to utilize the model predictions. Despite recent advances in machine modeling in the forecasting domain, there has been ample research in recent years showing that humans have a reluctance to embrace machine models. Clinicians are reluctant to rely on statistical methods for high stakes decisions, like mental health diagnoses, despite accumulated evidence showing the superiority of statistical methods across studies^24^. Forecasters put more weight on advice from human experts than statistical models, and discount models more when the same advice is labeled as a statistical prediction^25^. Algorithm aversion, a phenomenon where people punish machine models disproportionately to humans for making the same mistakes, can inhibit the adoption of machine models in forecast generation and may ultimately lead to suboptimal forecasts^26–28^.

Hybrid forecasting requires individuals to combine the model prediction with their beliefs. Our hybrid system (called SAGE^29^), allows forecasters to choose how to weight the model prediction to maximize the diversity required for the WOC effect. However, this freedom raises concerns about suboptimal and biased information processing. Specifically, individuals might overweight either the advice or their preexisting beliefs. Our system is designed to invoke the anchoring heuristic by exposing forecasters to a formalized, impartial baseline. However, anchoring can lead to systematic under-adjustment from the advice. Conversely, egocentric discounting is prevalent when individuals show a clear preference for congenial information. Confirmation bias is one such bias, resulting in overweighting confirmatory and discrediting conflicting information^30^.

### Advice taking

A key assumption in our hybrid system is that users will incorporate machine models with their own beliefs. Heeding advice tends to improve the accuracy of individual judgments and increases confidence^31,32^. Situational factors play a role in whether individual trust and use advice. The decision to use a piece of advice relies on both confidence in one’s beliefs and trust in the advice^33^. Generally, individuals overweight their own information and underweight the advice of others^34^. One concern is whether advice impacts how people form judgments. One study made advice available at different stages and found it has a smaller impact on research (termed “hypothesis generation”) than it has on judgment formation (termed “hypothesis testing”)^35^. Advice usage also changes with experience; as judges gain experience, they evaluate advice more carefully and are more likely to use it when it is helpful^34^.

Not surprisingly, judges evaluate advisers based on their expertise^32^. However, other factors influence the evaluation of advisers even when performance does not vary. Judges evaluate the experience of an adviser relative to themselves and only use advice when it achieves a perceived gain in expertise^34^. There is growing evidence that human judges view statistical advice differently than human advice. When the same information is described as either human or statistical, judges adjust less toward the statistical advice^25^. The reasons for human preference are debatable and may include an egocentric bias^36^, anchoring on pre-existing beliefs^37^, and better access to self or human justifications^38^.

### Social influence

In a hybrid system, it is also important to account for the influence users have on one another. Influence modeling is the body of work concerned with understanding how consensus is reached through the exchange of information among rational actors. Social influence can be beneficial or harmful within a large network. In forecasting, whether influence is beneficial to prediction is an open question. The benefit of social influence is a function of the group’s initial belief’s distance from the correct outcome^39^. When the group begins further away from the true outcome, the group benefits more from social influence. Consistent with our findings, earlier work shows a decreasing social influence effect as the distance from the initial beliefs increases^38^. However, social influence can also be harmful; it can produce herd-behavior or social loafing, decrease diversity, and lead to inaccurate forecasts^40^.

Most work on social influence is devoted to the study of influence coming from other (human) participants. Our model takes a new approach by quantifying the influence of independent machine forecasts. We build upon the DeGroot model^41^. The DeGroot model leverages a network, where nodes are individuals and edges indicate information sharing between individuals. The DeGroot model is an iterative model that, at each step, updates each individual’s belief by calculating a simple average among their neighbors. Several extensions to this model have been presented. For example^42^, one adjustment to the model considers the process by which opinions form^43^ provides an optimal solution to a specific type of game. Indeed, while a Bayesian information aggregation rule may be more appropriate, it’s not clear that agents would use such a complex computation. Further, there is experimental evidence supporting that the DeGroot model is a good approximation of the underlying aggregation process^44,45^.

### Quantifying machine influence

In this work, we study forecasts about geopolitical events. These forecasts are created on a hybrid forecasting platform^29^, Synergistic Anticipation of Geopolitical Events (SAGE), designed for this purpose. One of the key innovations of SAGE is that it allows forecasters to interact with computer-generated output during their process of generating forecasts. This computer-generated output can take the form of historical data pertaining to the question, or machine models that show a machine-generated prediction regarding its outcome. We assigned users to two conditions: a treatment condition where forecasters are exposed to machine models and a control condition where machine models are absent (both are shown in Fig. 5). In both conditions, participants saw historical data charts.

**Figure 1:**
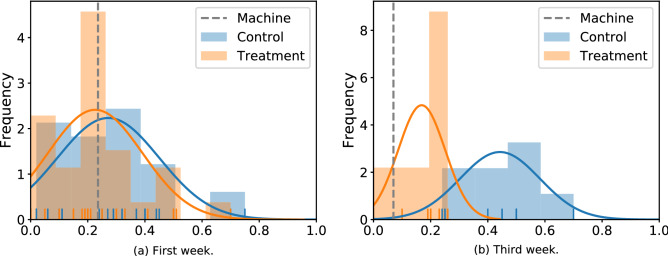
The figure shows the probability distribution projected on the correct option (hence, closer to one is more accurate) for the same question at two different time windows. The reference line represents the model prediction, and the colored histograms correspond to the two conditions. This example corresponds to the following question: “What will be the South Korean Won to one U.S. Dollar daily exchange rate on 29 June 2018?”.

Belief updating is a process in which individuals start with prior beliefs about a subject, and then update them as new information is made available. One widely used process for combining the priors and new information is the DeGroot model of belief updating^41^, described above. Despite its simplicity, DeGroot’s model generates accurate theoretical predictions of opinion formation in social networks^46,47^, and acts as a reasonable non-Bayesian one-step update rule^48^.

Here we use DeGroot’s model to quantify the influence of being exposed to machine forecasts when producing predictions on our platform. We assume that agent *i*’s forecast, $y_{i,t}$, at time *t* is a weighted combination of his prior belief $x_{i,t}$ and the current machine forecast $m_{t}$, i.e.,

1 $$\begin{aligned} y_{i,t} =\alpha _{i,t} x_{i,t}+(1-\alpha _{i,t})m_{t}, \end{aligned}$$

where $1-\alpha _{i}$ is the weight that the agent assigns to the machine forecast, and $\alpha$ determines the individual’s confidence in their own belief.

In many experimental studies of judge-advisor systems (JAS) with human advisors^38,49^ or algorithmic ones^50^, participants explicitly reveal their prior beliefs with the option to update them as new information is revealed. In our experimental design, agents’ prior beliefs are not directly observable, and thus, we need to infer them with a statistical approach. We use the control group, as opposed to individual forecasters, to estimate the prior beliefs of the group. We further assume the population is homogeneous, such that priors are normally distributed as $X_{i,t} \sim \mathcal {N}(\mu _{t},\Sigma _{t})$, where the mean and variance can be approximated from the realized forecasts of the control group. This with Equation (1) yields agents’ forecasts when exposed to machine models. This can be represented as

2 $$\begin{aligned} Y_{i,t}\sim \mathcal {N}(\alpha _t\mu _{t}+(1-\alpha _t)M_{t},\alpha _t^{2}\Sigma _{t}). \end{aligned}$$

The following example illustrates how influence perpetuates our system. Reviewing one specific question: “What will be the South Korean Won to one U.S. Dollar daily exchange rate on 29 June 2018?”. Naturally, new information about the price is available each day, which consequently can change the prior beliefs and the machine model predictions. Figure 1 depicts an example of the effects of being exposed to a machine forecast in one question, by comparing the distribution of forecasts between the control and treatment group at two different times. The figure shows the probability distribution projected on the correct option (hence, closer to one is more accurate) for one question at a specific time window. At the beginning (left figure), both the control and treatment group have similar distributions; as time goes by (right figure), the control group forecasts’ (i.e., prior beliefs) shift towards the correct option, however, participants exposed to the machine forecasts remain closer to the machine. This example shows a clear shift of mean and decrease of variance as a consequence of exposing participants to a reference forecast. The empirical evidence suggests that the weights participants put on the machine models are going to vary across time as a function of both the available information and the updated machine forecasts.

### Model estimation

 To estimate the weights on prior beliefs ($\alpha$), we train the model for each question using a rolling window using three different settings (see “Methods” section for more details). That is, for each question *q* we use maximum likelihood estimation to find $\alpha _{q,t}$ for each day *t*, using all the forecasts in the corresponding time window to infer the average weight $\alpha$ forecasters assign to their prior beliefs. Model estimation is detailed in the “Methods” section. We incorporate forecast skill into our model to account for interactions between highly and less engaged users. Knowledge and expertise plays a role in the differential discounting of advice^38^, and highly skilled forecasters interact with information and environments differently than typical forecasters^51^. To incorporate expertise in our analysis, we identify high and low skilled forecasters by assessing the prediction accuracy of users on a set of independent forecasting questions. Participants from both conditions also produced forecasts on a set of 126 independent questions, mostly categorical, that had no historical data or machine forecasts available—due to inaccessible data, impediments in ingesting data or response format. These independent items allow us to split the population into low (lower 50th percentile) and high (upper 50th percentile) levels of skill, based on participants’ accuracy in terms of normalized Brier scores (see definition of Brier score in Methods). We estimated a different prior for each of the two skill levels using the control group. This estimate enables us to quantify the machine influence uniquely for each question and subpopulation.

## Results

### Empirical validation of model

 Social influence studies on the wisdom of the crowds have shown that aggregated knowledge of individual information narrows the diversity of predictions without improvements in accuracy^40,52^. In our experimental setting, we have replaced “social influence” taking the form of an aggregate, with “machine influence” taking the form of a machine model prediction. The key difference is that machine forecasts are independent of the forecasts produced by the participants. Thus, we expect not only a decrease in diversity/variance, but a shift of the mean opinion when influenced by machine forecasts. Our model quantifies these two effects with a single parameter $\alpha$. Notice also that, based on Equation (2), the model always predicts a decrease of variance given by the relationship $\alpha ^2\sigma ^2_T=\sigma ^2_C$, where $\sigma ^2_T, \sigma ^2_C$ are the variance of the treatment and control condition, respectively. Indeed, when comparing the mean variance of forecasts between conditions across all questions, we find a significant (*p*-value $<3e-30$ with a t-test) decrease from $\sigma ^2_T=0.073$ to $\sigma ^2_C=0.052$.

### Cognitive hypotheses

Next, we analyze the observed weights to explore plausible cognitive mechanisms that drive the use and discounting of machine models and quantify their effects on the accuracy of users’ predictions. First, we present our hypotheses and in the following section, we present the results in a single analysis that addresses each hypothesis concurrently. We divide our hypotheses into two subcategories—strategies and biases—to analyze both the intentions and reactions of the users. First, we break down whether individuals intend to use the model and whether trust increases when the model is better. Then, due to the complexity of combining machine models with one’s prior beliefs, we cannot ignore the likelihood that estimates reflect biases in information processing. We test whether users are over-reliant on a) the model (anchoring) or b) their prior belief (confirmation bias).

#### User strategy

**Figure 2:**
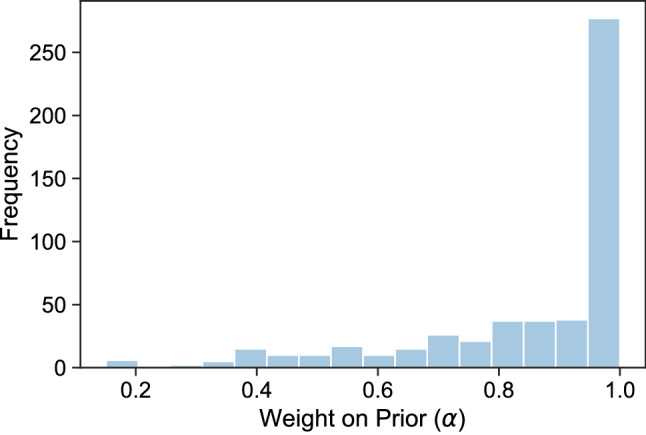
Distribution of the weight users put on their prior belief, αs. The average $\alpha$ is 0.86, where $\alpha = 1$ means users ignored the models, and $\alpha = 0$ means they used the model prediction as their own.

Do users trust the model predictions? Figure 2 shows the distribution of $\alpha$s, i.e. the weight participants put on their prior beliefs compared to machine predictions. The distribution is highly skewed towards 1 with an average $\alpha$ of 0.86, suggesting that participants are not very likely to incorporate the new information into their predictions. Such discounting of advice is consistent with prior work showing an “egocentric” bias in social settings^34,38,49^, where participants place similar high weights on their own opinion over others’ advice. The reputation of an adviser is another important indicator of trust in advice. It is easier to lose trust than it is to gain it^49^, and in a similar setting, assessors are sensitive to the accuracy and calibration of probabilistic forecasts^53^. Finally, we include difficulty into our model because advisees tend to overweight advice for difficult tasks and underweight advice for easy tasks^54^. We use the question’s difficulty based on the Brier scores (see Methods) of the control group forecasts. Brier scores are a measure of accuracy, which is naturally used to assess the quality of a probabilistic forecast. Our two primary hypotheses are that model’s trust should decrease (higher $\alpha$) for more difficult questions, and when the reputation of the models is lower. We define the reputation of a model as it’s average Brier score on all resolved questions.

#### Savviness

Are users savvy in choosing when to use the model? Can they perceive which models are more helpful? Our theoretical savviness hypothesis is that users will use the model when it performs better relative to the other human forecasters. We operationalize machine helpfulness as the percentage of people that had an inferior performance compared to the machine for a specific question and time window. We use the quantile metric^55^ to compare the performance of machine forecasts relative to the human forecasts, which essentially is the machine’s relative position on the cumulative distribution of the corresponding individual accuracy histogram (see Supplementary Figure S3). Our primary hypothesis is that we will observe greater model influence (a lower $\alpha$) when the model is more helpful (relative to humans). Our secondary hypothesis is that forecaster’s skill will interact with helpfulness, so better forecasters should trust the model more when it’s more helpful.

#### Anchoring effect

Anchoring is a judgmental bias describing how individuals under-adjust from arbitrary and random anchors when estimating a quantity, even when it is known that the anchor is arbitrary^56,57^. While there is debate about the mechanism(s) that produces anchoring effects, it is clear that increasing uncertainty and increasing effort decreases the impact of anchoring effects^58,59^. To quantify participant’s uncertainty we use variability (i.e. variance) of the group forecasts for a given question to measure the degree of consensus. Our primary hypothesis is that model influence should decrease (higher $\alpha$) for more uncertain questions. Our secondary hypothesis is that anchors will have an asymmetrical effect based on forecasting skill, so that better forecasters are more prone to adjust.

#### Egocentric discounting (confirmation bias)

Confirmation bias is the tendency to acquire or process new information in a way that confirms one’s preconceptions and avoids contradiction with prior beliefs^60^. Here we account for the possible asymmetric nature of confirmation bias on the differential weighting by users on the machine models. It is thus an open question to determine whether confirmation bias plays an important role in hybrid forecasting systems. Our primary hypothesis is that trust in the model should increase (lower $\alpha$) when the models confirms the prior beliefs of the participants.

The distance or discrepancy between priors and a reference opinion is also known to have an impact on the change of opinion^61^. Thus, we measure the interaction between the Euclidean distance and confirmation bias. Our secondary hypotheses are that (1) confirmation bias should be stronger for more skilled forecasters because more knowledge and experience is associated with stronger prior beliefs and greater egocentric discounting^30,38^; (2) confirmation bias should have an asymmetrical effect depending on the distance from the prior beliefs to the machine advice^62^.

#### Cognitive analysis

**Figure 3:**
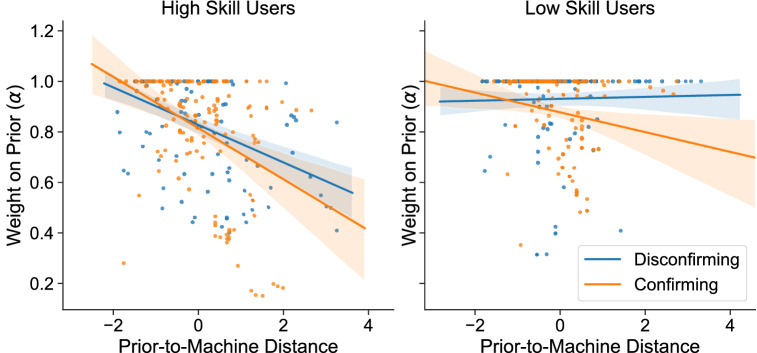
Relationship between weights $\alpha$s and the distance between prior beliefs and the machine forecasts. The *y*-axis is shared between the two plots. The left plot shows the relationship for the high-skill users, and the right plot for the low-skill users. The blue color depicts when the machine forecasts don’t confirm the prior beliefs, and the orange color shows when the machine forecasts confirm the priors of the group.

Three regression models predicting prior belief relative to machine influence ($\alpha$) are reported in Table 1. Our main dependent variable is the machine influence weight ($\alpha$). We use the predictor variables defined above to test our cognitive hypothesis adding potentially confounding variables such as the lifetime of a question. Model 1 includes only our primary hypotheses. Models 2 and 3 are extensions to test our secondary hypotheses. Model 2 includes interactions with forecaster skill to test our secondary hypothesis on savviness and anchoring effects. Egocentric discounting’s secondary hypothesis is tested in Model 3 by adding an interaction between distance from model to prior and confirmation bias. To minimize collinearity between the predictors, we mean-centered the measures by converting them to z-scores. We highlight the following findings:

- User strategy. The baseline level of trust in users’ priors for high-skill forecasters are less than the levels in low-skill forecasters. More skilled forecasters put more weight in the machine models than lower-skilled forecasters. In all three models, this is observed by comparing the skill (low and high) coefficients. We validate our hypothesis, finding forecasters trust their priors more and trust the machine advice less for more difficult questions. Moreover, we find trust is sensitive to the reputation of the model. When we account for changes in the model’s average performance (the model’s reputation), we find that users trust the model less after they see it perform poorly.
- Savviness. Forecasters are savvy enough to perceive when a machine model will be helpful. The negative coefficient for helpfulness indicates that as the machine models are more helpful, forecasters trust the models more. Moreover, there is evidence to suggest that high-skill users are savvier (approximately 4 times more) than low-skill users (see Model 2 in Table 1).
- Anchoring. The anchoring hypothesis indicates that increased uncertainty mitigates the effect size of model influence. The negative coefficients indicate that the relationship is reversed (i.e., more trust in the model for more uncertain questions). Regarding our secondary hypothesis, Model 2 highlights that better forecasters are more sensitive to the anchoring effects and higher uncertainty. Skilled users adjust more in the direction of the machine model as uncertainty increases.
- Confirmation bias. Our primary hypothesis on egocentric discounting is confirmed as trust in the machine model increases (i.e., lower $\alpha$) when the model confirms the prior beliefs of the forecasters. This is observed in the negative coefficients in all three models. Our secondary hypotheses are also validated by the significant interaction between confirmation, skill, and distance. Indeed, the effect size of confirmation bias is almost twice as large for low-skill forecasters. Second, we observe in Model 3, an asymmetric effect on distance when the model confirms the prior beliefs (distance$\times$confirming = $-0.057$***) compared to cases when it is disconfirming (distance = $-0.037$***). Figure 3 depicts the relationships between distance, confirmation bias and skill. The interaction effect with confirmation bias is greater for low-skill forecasters and mostly negligible for high-skill users. Yet, the effect of distance is greater for high-skilled forecasters. Thus, suggesting that high-skilled forecasters are more flexible but less biased by confirmation when assigning trust to the machine models, consistent with superforecasters’ tendency to revise their forecast frequently and incrementally^63^.

### Assessing the impact of cognitive biases

It is important to quantify the effect of the cognitive biases on the accuracy of the forecasts to assess the efficiency of our hybrid system. The main premise behind the SAGE system is that machine predictions will enhance human forecasters. However, machine models can harm the final forecasts if the human participants do not incorporate the advice from the machine models deliberately and effectively. Ecological rationality is achieved when the interaction between an environment and heuristics in decision-making enables effective behavior to be produced^64^. Providing end-users with objective evidence is not sufficient to assume it will be used well. Moreover, suboptimal decision processes can be overcome by the design of the decision structure. Thus, understanding barriers to how humans incorporate model predictions into their forecast is vital to unearthing possible policies and interventions that can increase the efficiency of the system.

**Table 1:** Regression analysis.

Variable	Model 1	Model 2	Model 3
Skill:high	0.848***	0.846***	0.842***
	(0.015)	(0.015)	(0.018)
Skill:low	0.932***	0.930***	0.943***
	(0.015)	(0.015)	(0.017)
Uncertainty	− 0.011		− 0.0181*
	(0.01)		(0.010)
Difficulty	0.021*		0.023**
	(0.012)		(0.012)
Confirming	− 0.058***	− 0.06***	
	(0.017)	(0.017)	
Helpfulness	− 0.031***		− 0.032***
	(0.010)		(0.010)
Lifetime	0.002	0.002	0.006
	(0.009)	(0.009)	(0.009)
Distance	− 0.060***	− 0.058***	− 0.037***
	(0.008)	(0.008)	(0.011)
Machine reputation	− 0.021**	− 0.021**	− 0.025***
	(0.009)	(0.009)	(0.009)
Interactions: distance $\times$ confirming			− 0.057***
			(0.017)
Interactions with skill helpful $\times$ skill:high		− 0.046***	
		(0.013)	
helpful $\times$ skill:low		− 0.015	
		(0.013)	
Uncertainty $\times$ skill:high		− 0.011	
		(0.013)	
Uncertainty $\times$ skill:low		− 0.003	
		(0.013)	
confirming $\times$ skill:high			− 0.044*
			(0.024)
confirming $\times$ skill:low			− 0.076***
			(0.023)
Observations	526	526	526
$\hbox {R}^{2}$	0.165	0.171	0.183
Adjusted $\hbox {R}^{2}$	0.152	0.155	0.167

The dependent variable is the percentage weight on human beliefs ($\alpha$). All independent variables were standardized, thus the coefficients describe how a one standard deviation change in a predictor variable affects the model influence. Binary predictors were dummy coded. Standard errors are displayed in parentheses.

***$$p<0.01$$; **$$p<0.05$$; *$$p<0.1$$.

**Figure 4:**
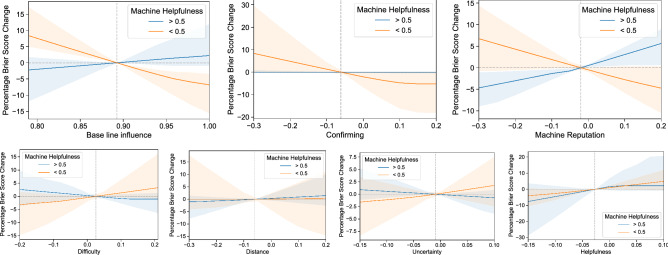
The impact of cognitive biases on forecast accuracy when exposed to high and low quality machine models. Each panel quantifies the impact (in terms of percentage Brier score difference) of exposing the control group to the machine models by changing the value of each of the coefficients (*x*-axis) in Eq. 3. The *y*-axis measures the difference in Brier scores, so positive values correspond to a decrease in accuracy, and negative values reflect an improvement. The colored lines show the medians; the shaded region depicts the interquartile range; the vertical line depicts the learned coefficient. The blue line depicts the impact on the questions that had high quality machine models, defined as those who performed better than 50% of individuals in the control group (i.e., “helpfulness” > 0.5); the orange line is the median change on questions with low quality machine models (i.e., “helpfulness”$\le 0.5$).

#### Impact of model influence

To asses whether the strategies and biases we observed are meaningful, we evaluate whether their impact is large enough to discriminate high-skill user’s from baseline influence. That is, we consider a factor to have a meaningful impact if its associated coefficient is greater than half the difference between the mean baseline influence and mean weight of high-skill users, $\approx 0.04$. The difference between our high and low skill users, $(Skill:low-Skill:high)$, represents a ceiling to the effect size that should outpace the impact of biases on the system because better forecasters consistently excel in ability and are less prone to harmful biases^51,63^. The results in Table 1 indicate that confirmation bias (operationalized by the “confirming” and “distance” variables) has a large effect on the weights in all models. Then, to a lesser extent, savviness (“helpfulness”) and trust (“machine reputation”) have a moderate impact on the model influence weights.

#### Impact on performance

To evaluate the the impact of cognitive biases on forecast accuracy, we use the behavioral patterns uncovered by our cognitive models in Table 1, to create a counterfactual framework to quantify the effects of the different biases. We use the following model trained on the data,

3 $$\begin{aligned} \alpha = \beta _1 + \beta _2 \; confirming + \beta _3 \; difficulty + \beta _4 \; distance + \beta _5 \; uncertainty + \beta _6 \; reputation + \beta _7 \; helpfulness \end{aligned}$$

to predict the respective human and machine influence weights ($\alpha$s) for each question and time window. Then, according to Equation 1, we use the learned weights to infer the final prediction of users exposed to machine models. In other words, based on the prior beliefs, we simulate how these beliefs change when being exposed to the machine model’s advice. We use our framework to quantify the difference in accuracy between two artificial treatment groups exposed to machine models with the same prior beliefs, by modulating the coefficients associated with each cognitive bias.

Figure 4 depicts the impact of cognitive biases by measuring the percentage accuracy difference between forecasts done with the learned model and those attained by changing the coefficients, respectively. We performed a parameter-based exploration of the $\beta$ coefficients in Equation 3, one at a time, to quantify the effect on forecast accuracy. We use percent change in Brier scores as a measure of gain in accuracy, so positive values mean a decrease of accuracy. The vertical lines in the figure depict the learned coefficient based on the data. Finally, under the ecological rationality framework, the impact of the biases depends on the environment where the decision are being made, which can be represented by the distribution of the machine models performance relative to the humans’ prior beliefs (see Supplementary Figure S3). Thus, we asses the impact of the biases differentiating between being exposed to low and high quality machine models based on the “helpfulness” variable. See Supplementary Figure S4 for the average impact on performance in our platform.

Looking at the confirmation bias and distance panels in Fig. 4, we first notice that, in contrast to confirmation bias having the most impact on the weights that humans assign to machines, their effect on the accuracy is marginal and most importantly, driven mostly by the low-quality models. The intuition behind this is that if a high-quality model is confirming someone’s prior beliefs, then both the model and the human prior must be accurate. However, when a low-quality model confirms the priors, then trusting the model can only decrease accuracy. Thus, we conclude confirmation bias in our environment is detrimental to performance. Next, we look into trust in the model based on machine reputation. As expected, increased trust in the models (i.e., a lower coefficient) as a function of their past performance only improves accuracy when the models are helpful. What is important to highlight is that the potential gains of trust in reputation coming from high-quality models are balanced by the losses of low-quality models. We see a similar effect for the baseline influence levels, although, less symmetrical as the impact of low-quality effects is higher. So, unless users have a strong belief that machine models are going to outperform humans, high levels of trust in humans priors, as we observed, are desired. In terms of savviness, we find that the strategies the human forecasters deployed are able to infer the relative “helpfulness” of machine models (given the negative coefficient), and increasing this competence would have a positive impact on performance. Finally, we observe that difficulty and uncertainty show minimal impact and room for improvement.

## Discussion

We investigate the problem of measuring how much trust human forecasters in a hybrid forecasting platform assign to a particular machine model. We develop a model that compares the forecasts of users who are exposed to the model with those who are not in order to learn the weight that the users assign to the model. Analyzing these weights helps uncover patterns about how humans interact with machine models throughout a forecasting tournament. Studying the trust between humans and machines reveals that users who can identify when the model is helpful are more accurate. We also leverage these weights to detect confirmation bias in our system which is detrimental only when machine models under-perform.

We divide our cognitive hypotheses into two categories, strategic and biases. From the strategic analysis, we find that users engage with machine model predictions similarly to how individuals use expert advice. They use their own information the majority of the time and only incorporate the advice in certain situations—when the task is difficult^3^. Only the best human forecasters intuitively recognize when the model is more helpful compared to other questions. Our impact analysis suggests there are interventions that could improve the performance of our system. When the machine model works well, the model reputation and relative helpfulness show the most room for improvement. This provides an opportunity to improve the system by providing information to the human forecasters about how well the model is expected to perform for a given question, such as based on the data source and question format, as well as highlighting the questions that seem to be most uncertain. We could also develop training based on these results to aid forecasters in how to assess the performance of a model prospectively.

From the biases analysis, we find that confirmation bias has a stronger effect than anchoring implying that forecasters are influenced more by their prior than by the model prediction. Where the machine models are suboptimal, cognitive biases are most detrimental to the system. In these cases, users would do better to trust their prior and only use the model when the question is highly uncertain. These improvements can be achieved by nudging users in the correct direction, via interventions or statistical debasing. On the other hand, it is difficult to mitigate the impact of deficient prior beliefs. A more realistic intervention would be to improve the reliability of priors. A forecasting system can be improved by encouraging, if not requiring, human forecasters to conduct comprehensive research prior to making a forecast. A hybrid system could further assist users in accessing better information. Priors could also improve with careful matching of forecaster expertise to question topics. Additionally, providing information about which questions are more difficult for the model relative to the humans would help encourage anchoring on the model when it is appropriate and to do more research when it is not.

This is a first step towards quantifying the impact of algorithms on forecast generation. We acknowledge some limitations of our approach and propose future research directions. First, we clarify that while DeGroot’s model may be unrealistically simple and naive about the learning process for individual agents, we use it to model the change of opinion in a population, not a single individual. Our empirical validations suggest the model is a good approximation of the average impact of being exposed to new information in the form of machine forecasts. Nevertheless, future work should be devoted to modeling the heterogeneity of the population, exploring better approximations using Bayesian inference techniques, and using different distributions as priors like the Dirichlet.

Our ability to track user behavior over a long-term forecasting tournament allowed us to quantify how model influence develops over time. Unfortunately, there are trade-offs with what could be achieved in a narrow, controlled environment. Primarily, it is difficult to decipher the difference between high confidence in the prior and low trust in the model. Both scenarios will produce an $\alpha$ close to 1.0. Additionally, we are unable to determine causality for some effects; notably, more engaged forecasters are better able to identify helpful models, or the ability to identify when a model is helpful makes a forecaster more accurate. We also cannot exclude that some factors deluge others, like we might have trouble detecting anchoring on the models because of the prevailing impact of confirmation bias. Future work should isolate the most relevant factors and biases in more targeted experimentation.

Finally, while our model is a major stride in quantifying model impact, we urge caution in generalizing to other settings. Research on trust in automation and algorithms often makes an assumption that all algorithms should be treated similarly. Trust in a model is governed by the ability of the model to serve the user’s needs. However, trust in humans and organizations is capricious and easily lost^65^. The results apply only to situations with similar model performance relative to humans. Appreciable changes to the structure or quality of models should alter trust in the models appreciably. Future research should explore how trust can be manipulated with increased transparency about the models’ past performance as well as studying the factors that affect how detectable model quality is to human judges.

As machine learning continues to progress, introducing data-driven algorithmic predictions to aid human decisions will become ubiquitous. For example, in bail decisions, large welfare gains are predicted by integrating machine predictions in the decision making process^66^. Algorithmic predictions are widely believed to outperform human predictions, and much attention has been devoted to the fact that humans fail to adjust their original judgments to incorporate the new information given to them. Yet, algorithms are far from perfect and do not necessarily beat humans in some deeply uncertain settings. Our results shed new light on the importance of accounting for situational factors that influence model reputation as well as biases in processing and aggregating evidence. In this setting, where model and human performance are comparable, the success of certain strategies and biases depends on their relative performance. The ability to detect when a model will be helpful is closely associated with the ability to make accurate forecasts, and overconfidence on algorithms may lead to detrimental results. A deeper understanding of the interaction of multiple cognitive biases is needed to build the path for human–machine hybrid decision systems.

## Methods

### Experimental design

The study was approved by the ethics committee of the USC University Park Institutional Review Board (USC UPIRB # UP-17-00527). Methods were carried out in accordance with relevant guidelines and regulations. Subjects gave written informed consent prior to participation. Participants were recruited by the Intelligence Advanced Research Projects Activity (IARPA) HFC Test and Evaluation Team. Participants participated on a voluntary basis and did not receive compensation for their participation.

All of the forecasting problems in SAGE take the following form. First, each question pertains to a quantity. For example, the question pertains to a price prediction of Japan’s Nikkei 225 index. A list of all questions can be seen in Supplementary Table S2. Second, there is a time span for the questions to be answered. For a given question, these times are fixed for all users across the system. Third, the resolution criteria provides the user with an explicit link to the piece of evidence that will be used to decide the correct answer to the question, in this example Google Finance. Finally, users enter their forecasts by assigning a probability to a set of answer options. Each question contains between 2 to 5 answer options, inclusive. The answer options are non-overlapping and are generated based upon historical data values for the quantity in question. Users enter their forecasts by assigning a probability to each answer option. When the question closes, exactly one answer option is selected as the correct answer.

**Figure 5:**
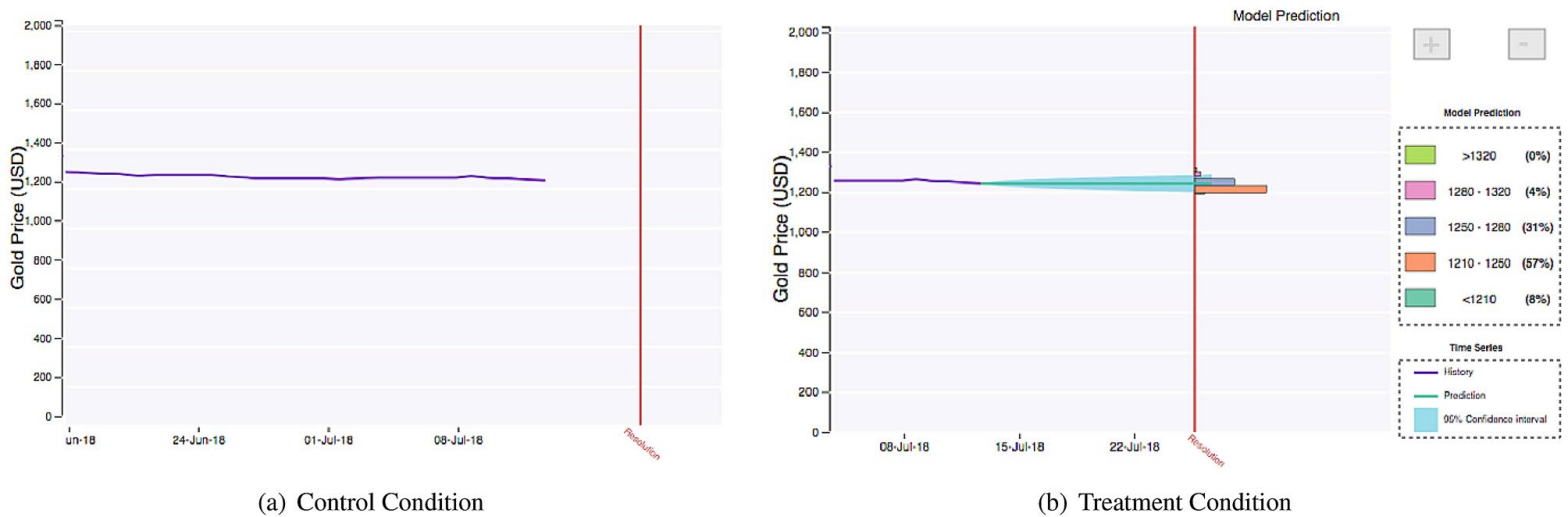
Comparison of the information displayed in the two conditions in the SAGE platform on the same question. Panel A is our control condition which had access to historical data charts. Panel B is our experimental condition which also had access to machine model predictions.

In Fig. 5a, we see information shown in the *control condition*. In this condition, users are exposed to the historical data of the quantity that they are asked to forecast in the form of a chart. The chart shown in the figure is the only information they are shown; no additional information such as summary statistics are provided. Furthermore, this chart is exactly what users on the site see.

Figure 5b shows additional information shown in the *treatment condition*. Here, users see all of the information in the control condition, but the historical data is augmented with a machine prediction. The green line shows the machine model’s prediction about the quantity, and the shaded green region around it is the 95% confidence interval. This additional information can be used by the forecasters in generating their prediction, but it is not required or encouraged beyond the display of this plot.

### User recruitment

The participants of the study were recruited through public recruitment carried out on blogs, web sites, and other geopolitical forecasting platforms. Each member of this outsourced pool of people was randomly assigned to either the treatment or control condition. Recruitment was done exclusively before the beginning of the study. The study lasted approximately 7 months, in which new questions were added to the platform each week. Participation was unpaid and voluntary and relied purely on the engagement of users. Engagement was encouraged through the use of weekly emails notifying users of new questions. On the platform itself, a leaderboard was maintained to give credit to users who made accurate forecasts. Supplementary Table S1 shows a summary of the participant in each of the conditions and the number of total predictions made. Supplementary Figures S1–S2 depicts the demographic characteristics of the participants. We conducted a survival analysis to confirm that users in each condition showed no significant difference in attrition (see Supplementary Figure S5). Providing access to machine models did not influence engagement meaningfully.

### Machine predictions

Given that questions covered a broad set of geopolitical topics and different data sources, we used a general approach to forecast time series. Specifically, we use the AutoRegressive Integrated Moving Average (ARIMA) model to produce machine forecasts from the historical data relevant to each question. The parameter selection for the ARIMA model was done automatically using a standard R-package described in^67^. Recently, the M4 competition measured the performance of machine models across over 100,000 problems, many related to the geopolitical events discussed in this paper. They found that general models, like the ARIMA one used in this work, perform the best across a wide array of problems, even in comparison to more complex machine learning models^68,69^.

While ARIMA is often used for obtaining pointwise estimates, here we need to generate probabilistic forecasts over the possible answers, i.e., the answer options shown in Fig. 5b. We do this by calculating the prediction intervals under the somewhat standard assumption that the residuals are normally distributed and uncorrelated with each other. The resulting probabilistic forecasts are then shown to the human forecaster as indicated in Fig. 5b.

As new data for an active question became available, or existing data was updated, we updated the machine models with the latest data. This is done so that, in the treatment condition, the machine model that is presented to the users is based on the most recent version of the data. In both conditions, this is done so that the forecasters can make an informed forecast using the latest historical data.

An important note about our machine models. Most of the experimental work in the literature assume that machine models are superior to humans^26,50^. This is not true in this work. Due to the difficulty in predicting outcomes of geopolitical events, there are problems where machines under-perform compared to humans. Part of our analysis will focus on humans’ ability to identify models that performed poorly.

### Model estimation

Here, we formalize how to quantify the average weight that a population of forecasters puts on the machine models. Let $X_{i,t},Y_{i,t}$ be the prior and realized forecast of agent *i* at time *t*. Agent *i* arrives and his forecast is influenced by the current machine forecast $M_{t}$ as follows,

$$\begin{aligned} Y_{i,t}&=\alpha X_{i,t}+(1-\alpha )M_{t}\\ X_{i,t}&\sim \mathcal {N}(\mu _{t},\Sigma _{t}), \end{aligned}$$

where the key idea is to use the control group to estimate the group prior beliefs $X_{t}$. Thus, agents’ forecasts when exposed to machine models follows the following distribution,

$$\begin{aligned} Y_{t}\sim \mathcal {N}(\alpha \mu _{t}+(1-\alpha )M_{t},\alpha ^{2}\Sigma _{t}). \end{aligned}$$

We can now formulate the probability density, under the model, of seeing data $(y_{1,}m_{1}),(y_{2},m_{2}),\ldots (y_{n},m_{n})$,

$$\begin{aligned} \prod _{i=1}^{n}p\left( y_{i}|m_{i};\alpha ,\mu ,\Sigma \right) = \prod _{i=1}^{n}\frac{\exp \left( -\frac{1}{2}V^{T}(\alpha ^{2}\Sigma )^{-1}V \right) }{\sqrt{(2\pi )^{k}\alpha ^{k}|\Sigma |}}, \end{aligned}$$

where $V=(y_{i}-\alpha \mu _{t}-(1-\alpha )m_{t})$. Then, to estimate the parameter $\alpha$ for each question, we use a non-linear optimization algorithm to maximize the log-likelihood. To avoid problems with the sparseness of data and singular matrices, we transform questions with multiple answer options into a binary question where the two answer options correspond to the probability of the correct option and the probability of the incorrect option, respectively.

### Time window length

Given that the duration of the questions varies greatly (mean is 53 days and standard deviation is 33) and longer questions have sparser forecasts, we use three different settings for the length of the time window when estimating on each question. Time windows with less than four forecasts per condition were discarded. Our three approaches consisted of: (1) Using a weekly rolling window; (2) Utilizing a rolling window of length equal to one fourth of the duration of each question. This setting is useful for long questions that have sparse forecasts; (3) Applying the maximum between weekly or quarterly time windows. Results presented here are using the last approach, however, the conclusions are consistent across all three approaches (Supplementary Figures S6–S8 depict the results for all three settings).

### Independent variables

For evaluating accuracy, we extend the notion of Brier scores^70^ to account for ordinal questions. To address the fact that some forecasting questions have ordered outcomes, for example predicting the price of the Nikkei, we use a variant of the Brier score that uses the cumulative probabilities instead of the densities^71^.

#### Definition 1

(Brier Score). Given a question of duration *T*, forecasts $p_t=(p_{i,t},p_{2,t},\ldots ,p_{n,t})$, $1\le t\le T$, and the actual outcome $o\in \Delta ^n$, we use the Brier score *B*(*p*) as a measure of accuracy:

$$\begin{aligned} B(p)=\frac{1}{T}\sum _{t=1}^T\sum _{i=1}^n\left( p_{i,t} -o_i \right) ^2. \end{aligned}$$

We define a dummy variable, ’*confirming*’, to represent whether the machine is supporting the beliefs of the group according to Definition 2 or not.

#### Definition 2

(Confirmation Bias). Let $p=(p_1,p_2,\ldots ,p_n)$ be a probability forecasts, we define $x=(x_1,x_2,\ldots ,x_n)\in \Delta ^n$ as the closest extreme (i.e., where all the probability mass is assigned to one option). Formally,

$$\begin{aligned} x(p)=\arg \min _{x\in \Delta ^n} ||x-p ||_2 \end{aligned}$$

We define a machine forecast *m* to be confirming one’s prior beliefs if the new information is closer to your extreme probability than your priors, i.e.,

$$\begin{aligned} ||x-m ||_2 \le ||x-p||_2 \end{aligned}$$

**Skill**: Dummy variable representing if a user belongs to the top or lower 50th percentile on a set of independent forecasting questions.
**Uncertainty**: Defined as the average of the diagonal elements in the covariance matrix for users forecasts from the control group.
**Difficulty**: Average Brier score of users’ forecasts from the control group (i.e., priors beliefs ).
**Confirming**: Confirmation Bias (Definition 2) between the model’s forecasts and the mean forecast from the treatment group.
**Confirming**: Dummy variable representing the confirmation bias (Definition 2) between the model’s forecasts and the mean forecast from the treatment group.
**Helpfulness**: Defined as the percentage of users from the control group that had an inferior performance compared to the machine model.
**Lifetime**: The percentage of days out of the total that a question has been open for forecasts.
**Distance**: Average Euclidean distance, using the cumulative probabilities, between the machine model’s forecasts and the users’ forecasts from the control group.
**Machine reputation**: Past performance of the machine models. Defined as the negative of the average Brier Score of the machine forecasts for questions that had resolved by the beginning of the current question.

## Supplementary information

Supplementary information.

## Data Availability

The data that support the findings of this study are available from the corresponding author upon request.
