# Supplementary material for: Quantifying machine influence over human forecasters
Source: Sci Rep. 2020 Sep 29;10:15940. doi: 10.1038/s41598-020-72690-4 (PMC7524768; doi:10.1038/s41598-020-72690-4)
Supplement: Supplementary file 1 — Supplementary information. [file 41598_2020_72690_MOESM1_ESM.pdf]

Supplementary Information

Quantifying Machine Influence over Human Forecasters

Andrés Abeliuk[1,*], Daniel Benjamin[1], Fred Morstatter[1], and Aram Galstyan[1]

[1]Information Sciences Institute, University of Southern California, Marina del Rey, CA, USA.
[*]Correspondence to aabeliuk@isi.edu

S1 Supplementary Tables

Table S1: Summary of participants and forecasts for each condition.

Condition	Participants	Forecasts	Questions
Control	96	2152	62
Treatment	192	2217	62

Table S2: Questions and their respective category.

Question	Category
What will be the daily closing price of gold on 5 September 2018 in USD?	Macroeconomics/Finance
What will be the daily closing price of Japan's Nikkei 225 index on 5 September 2018?	Macroeconomics/Finance
What will be the daily closing price of gold on 29 August 2018 in USD?	Macroeconomics/Finance
How many battle deaths will ACLED record in South Sudan in August 2018?	Politics/Intl Relations
How much crude oil will Nigeria produce in July 2018?	Macroeconomics/Finance
What will be the daily closing price of gold on 22 May 2018 in USD?	Macroeconomics/Finance
What will be the short-term interest rate for Indonesia (IDN) in July 2018?	Other
What will be the FAO Sugar Price Index in June 2018?	Macroeconomics/Finance
How much crude oil will Iraq produce in May 2018?	Macroeconomics/Finance
Will ACLED record any civilian fatalities in Iran in August 2018?	Politics/Intl Relations
How much crude oil will Saudi Arabia produce in July 2018?	Macroeconomics/Finance
How many 'hacking or malware (HACK)' data breaches will Privacy Rights Clearinghouse record in July 2018?	Technology
How many battle deaths will ACLED record in Central African Republic in August 2018?	Politics/Intl Relations
Will ACLED record any riot/protest events in Cameroon in June 2018?	Politics/Intl Relations
What will be the daily closing price of Brent crude oil (USD per barrel) on 31 July 2018, according to the U.S. EIA?	Macroeconomics/Finance
What will be the daily closing price of Swiss Market Index (INDEXSWX: SMI) on 28 June 2018?	Macroeconomics/Finance
Will ACLED record any riot/protest events in Gambia in July 2018?	Politics/Intl Relations

Continued on next page

Table S2 – *Continued from previous page*

Question	Category
What will be the daily closing price of Brent crude oil (USD per barrel) on 16 August 2018, according to the U.S. EIA?	Macroeconomics/Finance
Will ACLED record any civilian fatalities in Sri Lanka in July 2018?	Politics/Intl Relations
What will be the approval rate for Japan's cabinet in NHK's monthly survey in June 2018?	Politics/Intl Relations
How many earthquakes of magnitude 5 or stronger will occur worldwide in May 2018?	Natural Sciences/Climate
How many battle deaths will ACLED record in Ethiopia in August 2018?	Politics/Intl Relations
What will be the daily closing price of gold on 27 July 2018 in USD?	Macroeconomics/Finance
Will ACLED record any civilian fatalities in Burkina Faso in August 2018?	Politics/Intl Relations
What will be the FAO Dairy Price Index in May 2018?	Macroeconomics/Finance
How many earthquakes of magnitude 6 or stronger will occur worldwide in July 2018?	Natural Sciences/Climate
How many deaths perpetrated by Boko Haram will the Council on Foreign Relations report for July 2018?	Politics/Intl Relations
What will be the maximum sea ice extent on the Bering Sea between 14 March 2018 and 10 April 2018?	Other
What will be the long-term interest rate for Russia (RUS) in June 2018?	Macroeconomics/Finance
What will be the short-term interest rate for Canada (CAN) in July 2018?	Other
What will be the closing value of the Mexican Peso to one U.S. Dollar exchange rate on 6 July 2018?	Other
How many 'hacking or malware (HACK)' data breaches will Privacy Rights Clearinghouse record in May 2018?	Technology
What will be the daily closing price of gold on 5 June 2018 in USD?	Macroeconomics/Finance
What will be the daily closing price of the EURO STOXX 50 Index (SX5E) on 29 August 2018?	Macroeconomics/Finance
What will be the daily closing price of France's CAC 40 index (INDEXEURO: PX1) on 31 August 2018?	Macroeconomics/Finance
How many battle deaths will ACLED record in Yemen in May 2018?	Politics/Intl Relations
What will be the daily closing price of gold on 26 April 2018 in USD?	Macroeconomics/Finance
How much crude oil will Libya produce in May 2018?	Macroeconomics/Finance
What will be the FAO Cereal Price Index in August 2018?	Macroeconomics/Finance
What will be the long-term interest rate for South Africa (ZAF) in July 2018?	Macroeconomics/Finance
How much crude oil will Venezuela produce in July 2018?	Macroeconomics/Finance
What will be the short-term interest rate for India (IND) in July 2018?	Other
What will be the daily closing price of Brent crude oil (USD per barrel) on 10 July 2018, according to the U.S. EIA?	Macroeconomics/Finance
What will be the long-term interest rate for Hungary (HUN) in July 2018?	Macroeconomics/Finance
How many deaths perpetrated by Boko Haram will the Council on Foreign Relations report for June 2018?	Politics/Intl Relations
What will be the monthly period-over-period change in the consumer price index (CPI) for Egypt in May 2018?	Macroeconomics/Finance
What will be the monthly period-over-period change in the consumer price index (CPI) for Benin in April 2018?	Macroeconomics/Finance
Will ACLED record any riot/protest events in Gabon in April 2018?	Politics/Intl Relations
What will be the daily closing price of Brent crude oil (USD per barrel) on 31 May 2018, according to the U.S. EIA?	Macroeconomics/Finance
What will be the short-term interest rate for the Czech Republic (CZE) in June 2018?	Macroeconomics/Finance
What will be the daily closing price of the United Kingdom's FTSE 100 Index (INDEXFTSE: UKX) on 22 August 2018?	Macroeconomics/Finance
What will be the long-term interest rate for Greece (GRC) in March 2018?	Macroeconomics/Finance
What will be the South Korean Won to one U.S. Dollar daily exchange rate on 29 June 2018?	Macroeconomics/Finance
What will be the long-term interest rate for Portugal (PRT) in April 2018?	Other
How many battle deaths will ACLED record in Afghanistan in April 2018?	Politics/Intl Relations
How many United Nations Security Council Resolutions concerning Syria will be vetoed by Russia between 22 April 2018 and 22 August 2018?	Politics/Intl Relations

Continued on next page

Table S2 – *Continued from previous page*

Question	Category
Will ACLED record any civilian fatalities in Ghana in June 2018?	Politics/Intl Relations
Will ACLED record any riot/protest events in Egypt in August 2018?	Politics/Intl Relations
How many material conflict events involving Occupied Palestinian Territory will ICEWS record in March 2018?	Politics/Intl Relations
What will be the maximum sea ice extent on the Baffin Bay Gulf of St. Lawrence between 21 March 2018 and 10 April 2018?	Other
How many earthquakes of magnitude 5 or stronger will occur worldwide in March 2018?	Natural Sciences/Climate
How many 'hacking or malware (HACK)' data breaches will Privacy Rights Clearinghouse record in April 2018?	Technology

S2 Supplementary Figures

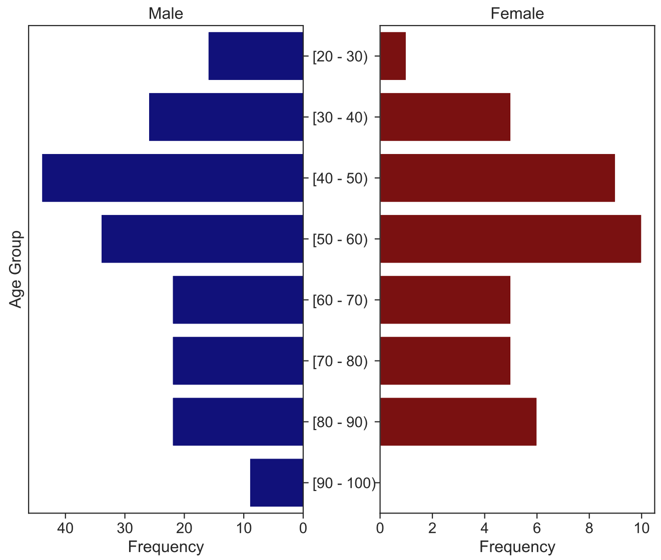

Figure S1: Gender and age demographics of participants.

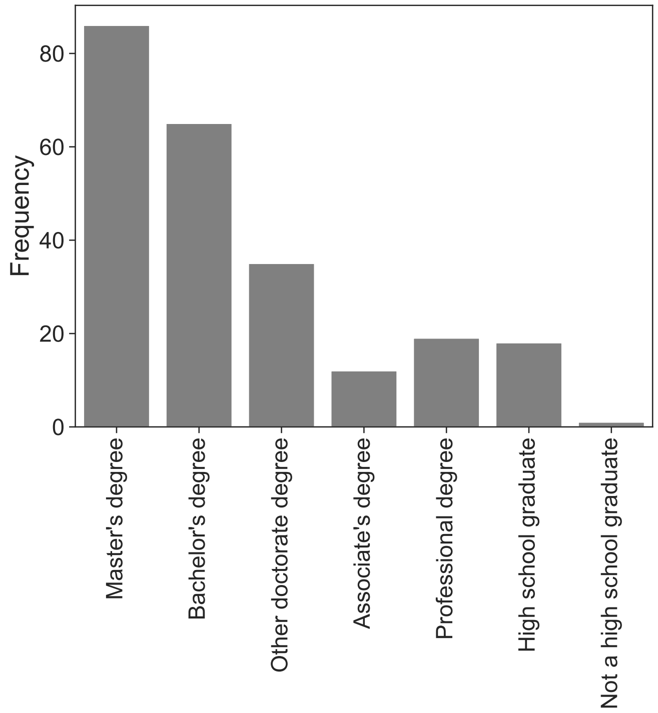

Figure S2: Education demographics of participants.

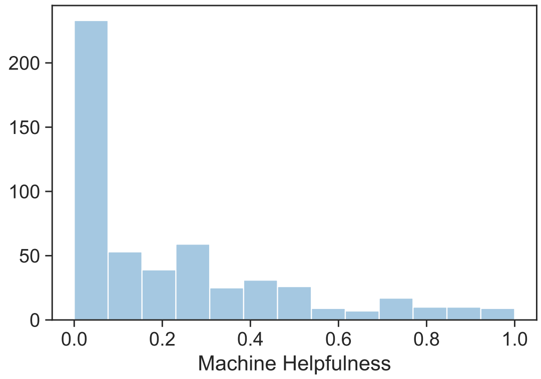

Figure S3: Distribution of machine helpfulness variable. Defined as the percentage of users from the control group that had an inferior performance compared to the machine model.

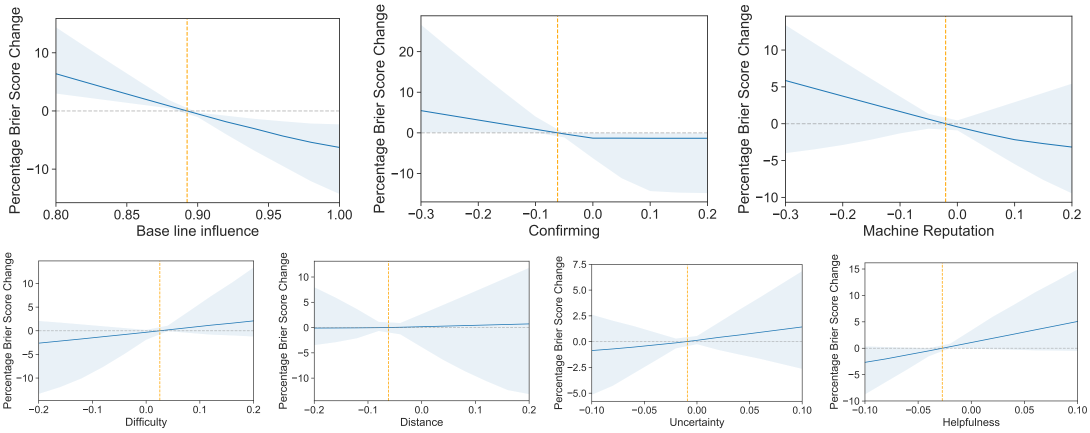

Figure S4: The impact of cognitive biases on accuracy. Each panel quantifies the impact (in terms of percentage Brier score difference) of exposing the control group to our machine models by changing the value of each of the coefficients (x-axis) of the linear regression model. Y-axis measures the difference in Brier scores, so positive values correspond to a decrease in accuracy and negative values reflect and improvement. The bold line is the median; the shaded region depicts the interquartile range; the vertical line depicts the learned coefficient.

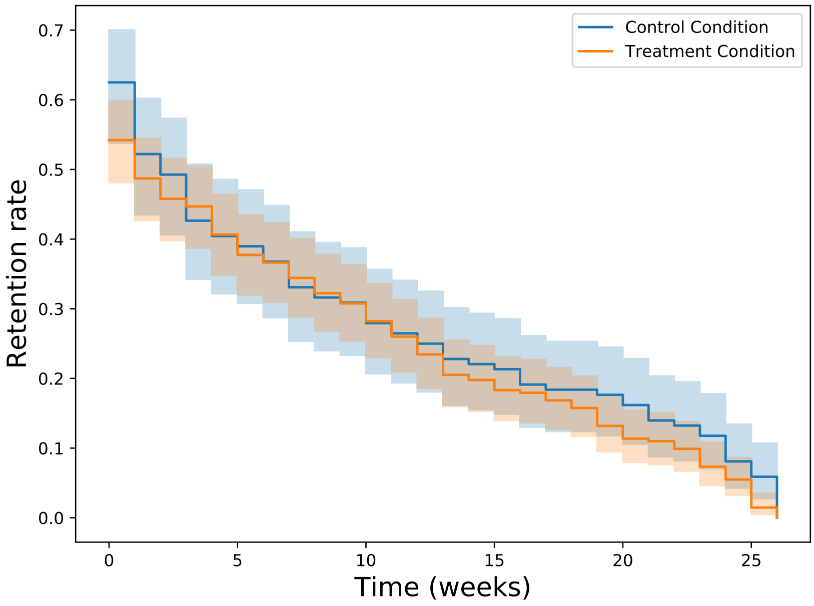

Figure S5: Kaplan-Meier estimated user retention rates for each condition. Error bars depict 95% confidence intervals. Both conditions show no significant differences in retention rates.

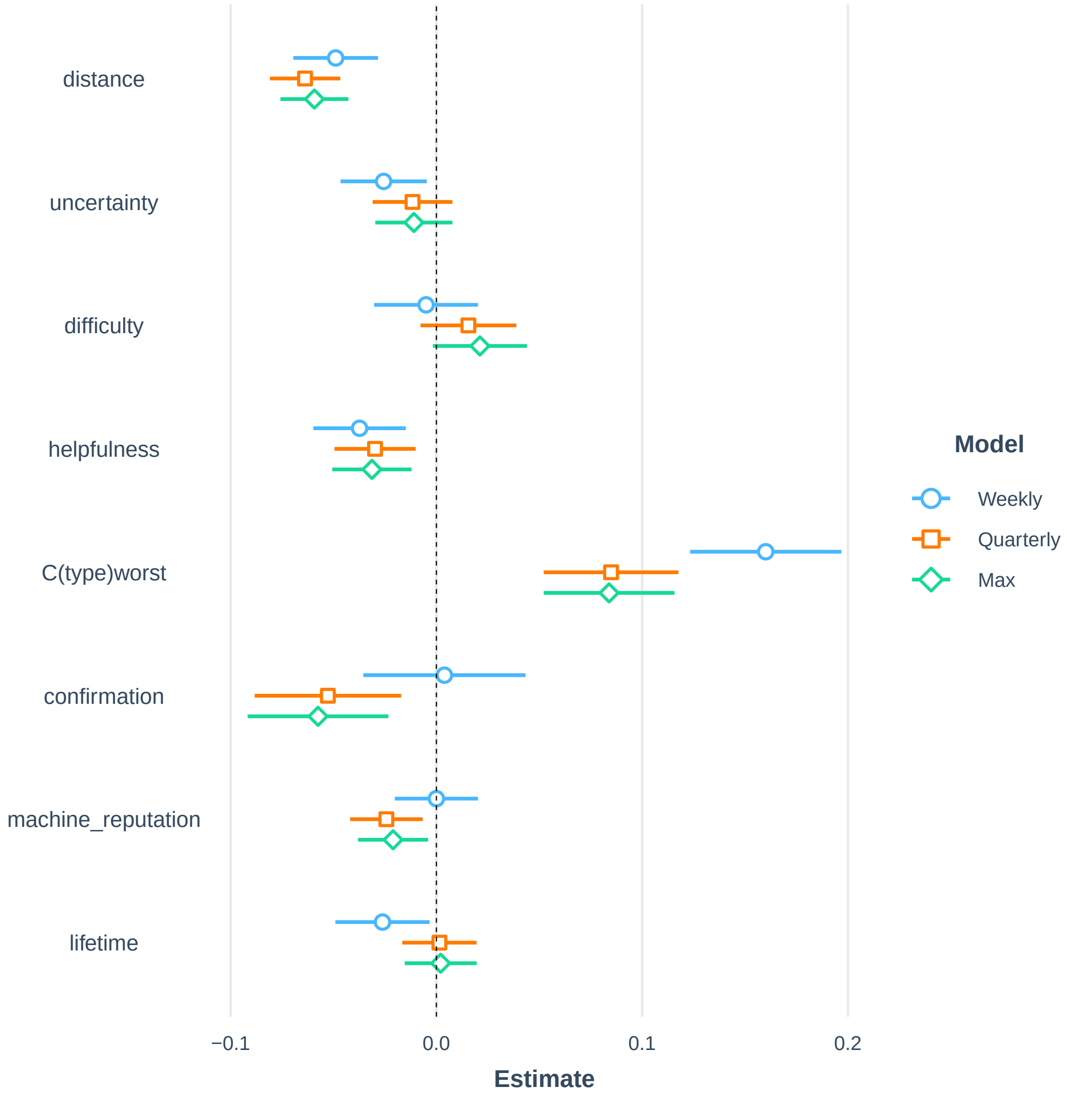

Figure S6: Sensitivity analysis of regression Model 1 for different time window parameters. Error bars depict 95% confidence intervals of estimated coefficients. Weekly model (blue) uses a weekly rolling window. Quarterly (orange) uses a rolling window of length equal to a fourth of the duration of each question. This setting is useful for long questions that have sparse forecasts. Max model uses the maximum between weekly or quarterly time windows. The corresponding R^2 and number of observations for each model are: 1) Weekly $R^2 = 0.377$, No. Observations = 262; 2) Quarterly $R^2 = 0.168$, No. Observations = 512; 3)Quarterly $R^2 = 0.165$, No. Observations = 526.

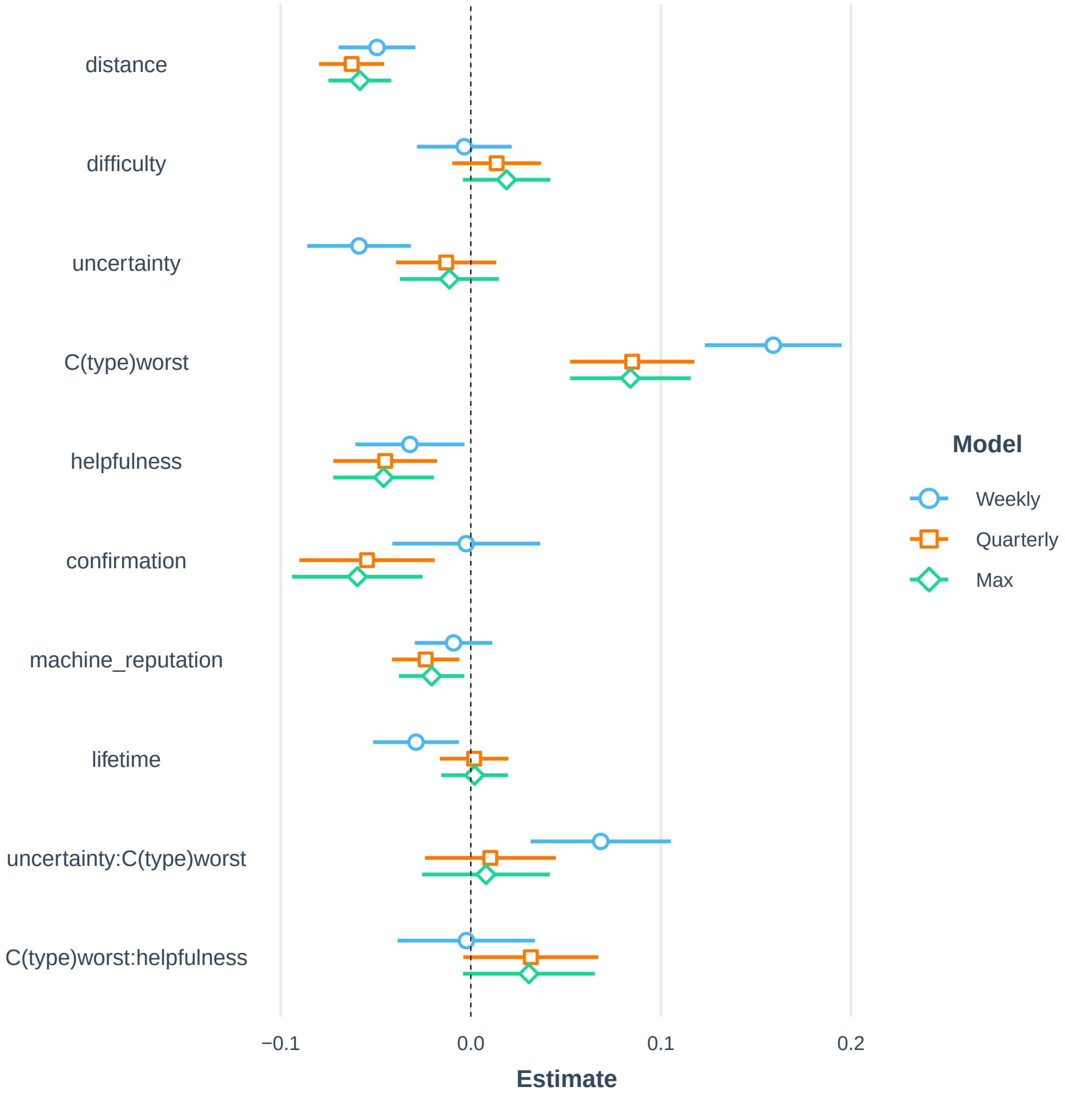

Figure S7: Sensitivity analysis of regression model 2 for different time window parameters. Error bars depict 95% confidence intervals of estimated coefficients. Weekly model (blue) uses a weekly rolling window. Quarterly (orange) uses a rolling window of length equal to a fourth of the duration of each question. This setting is useful for long questions that have sparse forecasts. Max model uses the maximum between weekly or quarterly time windows. The corresponding R^2 and number of observations for each model are: 1) Weekly $R^2 = 0.409$, No. Observations = 262; 2) Quarterly $R^2 = 0.176$, No. Observations = 512; 3)Quarterly $R^2 = 0.171$, No. Observations = 526.

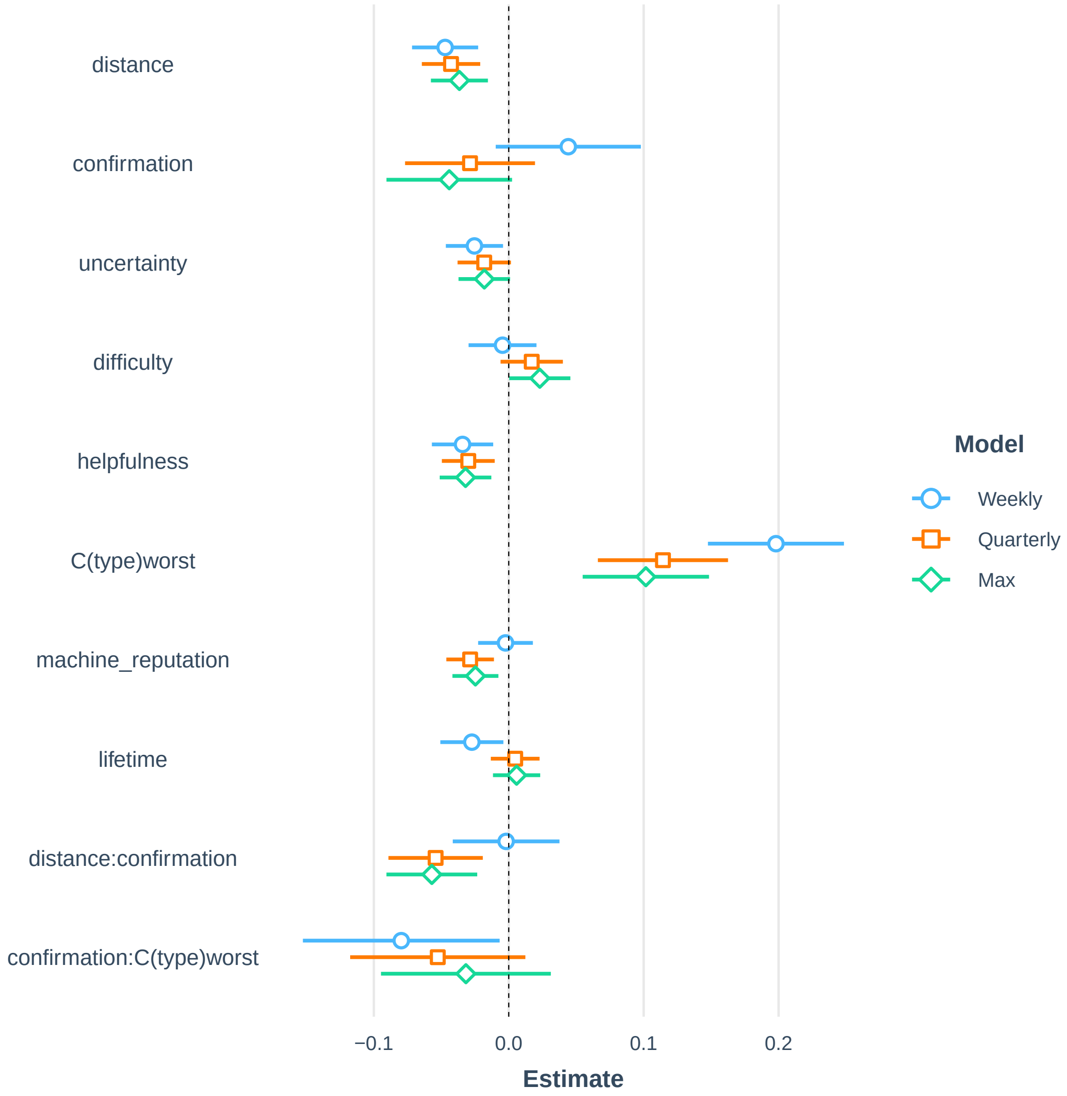

Figure S8: Sensitivity analysis of regression Model 3 for different time window parameters. Error bars depict 95% confidence intervals of estimated coefficients. Weekly model (blue) uses a weekly rolling window. Quarterly (orange) uses a rolling window of length equal to a fourth of the duration of each question. This setting is useful for long questions that have sparse forecasts. Max model uses the maximum between weekly or quarterly time windows. The corresponding R^2 and number of observations for each model are: 1) Weekly $R^2 = 0.388$, No. Observations = 262; 2) Quarterly $R^2 = 0.187$, No. Observations = 512; 3)Quarterly $R^2 = 0.183$, No. Observations = 526.
